# Use of Cyclic Backbone NGR-Based SPECT to Increase Efficacy of Postmyocardial Infarction Angiogenesis Imaging

**DOI:** 10.1155/2017/8638549

**Published:** 2017-10-24

**Authors:** Geert Hendrikx, Tilman M. Hackeng, Rick van Gorp, Matthias Bauwens, Leon J. Schurgers, Felix M. Mottaghy, Mark J. Post, Ingrid Dijkgraaf

**Affiliations:** ^1^Department of Radiology and Nuclear Medicine, Cardiovascular Research Institute Maastricht (CARIM), Maastricht University Medical Center (MUMC+), Maastricht, Netherlands; ^2^Department of Biochemistry, Cardiovascular Research Institute Maastricht (CARIM), Maastricht University Medical Center (MUMC+), Maastricht, Netherlands; ^3^Department of Nuclear Medicine, University Hospital, RWTH University, Aachen, Germany; ^4^Department of Physiology, Cardiovascular Research Institute Maastricht (CARIM), Maastricht University Medical Center (MUMC+), Maastricht, Netherlands

## Abstract

As CD13 is selectively expressed in angiogenesis, it can serve as a target for molecular imaging tracers to noninvasively visualize angiogenic processes in vivo. The CD13-targeting moiety NGR was synthesized and cyclized by native chemical ligation (NCL) instead of disulfide bridging, leading to a cyclic peptide backbone: cyclo(Cys-Asn-Gly-Arg-Gly) (coNGR). Beside this new monomeric coNGR, a tetrameric NGR peptide co(NGR)_4_ was designed and synthesized. After radiolabeling, their in vitro and in vivo characteristics were determined. Both coNGR-based imaging agents displayed considerably higher standardized uptake values (SUVs) at infarcted areas compared to the previously reported disulfide-cyclized cNGR imaging agent. Uptake patterns of ^111^In-coNGR and ^111^In-co(NGR)_4_ coincided with CD13 immunohistochemistry on excised hearts. Blood stability tests indicated better stability for both novel imaging agents after 50 min blood incubation compared to the disulfide-cyclized cNGR imaging agent. In mice, both coNGR peptides cleared rapidly from the blood mainly via the kidneys. In addition, co(NGR)_4_ showed a significantly higher specific uptake in infarcted myocardium compared to coNGR and thus is a promising sensitive imaging agent for detection of angiogenesis in infarcted myocardium.

## 1. Introduction

Angiogenesis is an endogenous healing process which serves to restore tissue blood supply in response to ischemic injury [1]. The extent of angiogenic activity is correlated with infarct healing and postmyocardial infarction (MI) remodeling [2]. Augmentation of experimental angiogenesis has several beneficial effects on post-MI remodeling, including reduced apoptosis of hypertrophic cardiomyocytes in the border zone, attenuated collagen deposition and scar formation in the noninfarcted zone, and improved long-term ventricular function [2, 3]. However, while positive results were shown in animal models of MI, the benefit for MI patients has yet to be shown. So far, results from double-blind placebo-controled trials were disappointing [4–7]. These negative outcomes were most likely attributable to a combination of factors, including patient selection, choice of delivery platforms for therapeutic agents, and, importantly, lack of sensitive noninvasive detection methods for angiogenesis [8].

Frequently employed imaging agents for noninvasive nuclear imaging of cardiovascular angiogenesis in animal models are based either on the *α*_v_*β*_3_ integrin-targeting Arg-Gly-Asp (RGD) amino acid sequence or on vascular endothelial growth factor (VEGF) [9]. Several single-photon emission computed tomography (SPECT) and positron emission tomography (PET) based studies reported positive results with RGD targeting agents (reviewed in [10, 11]). However, in competition studies, the RGD motif was found to have a lower target homing ratio (target to control tissue) compared to the Asn-Gly-Arg (NGR) tripeptide sequence which binds to selectively upregulated CD13 on angiogenically active endothelial cells [12]. Given the high target homing ratio of cyclic NGR peptides, employing a cyclic NGR-based imaging agent might result in better image quality.

The CD13-targeting NGR motif has been explored at our institute as a molecular angiogenesis imaging agent for fluorescence microscopy [13], magnetic resonance imaging (MRI) [14], and more recently single-photon emission computed tomography (SPECT) imaging [15]. These cyclic NGR-based molecular imaging agents showed enhanced uptake in the infarct area and infarct border zone 7 days after MI in a mouse model [13–15]. Although each study established specific binding of their respective NGR-based ligand in the infarct area and infarct border zone, none of these molecular imaging agents had the potential for clinical translation at this stage. While localizing the fluorescently labeled NGR-based molecular imaging probe would require an invasive procedure, the cadmium-selenium core of cyclic NGR-conjugated paramagnetic quantum dots (pQD) in combination with their accumulation in liver and spleen hampers translation of the MRI angiogenesis imaging agent into clinical trials. For the NGR-based SPECT tracer to be considered as a potential clinical angiogenesis imaging tool, specific target uptake has to increase.

**Figure 1:**
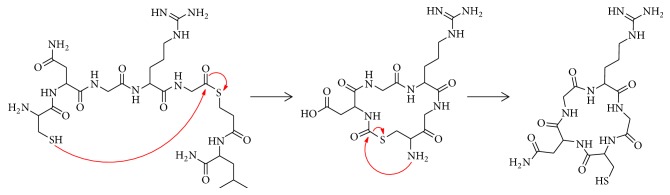
Reaction mechanism of cyclization of CNGRG-MpaL via NCL.

**Figure 2:**
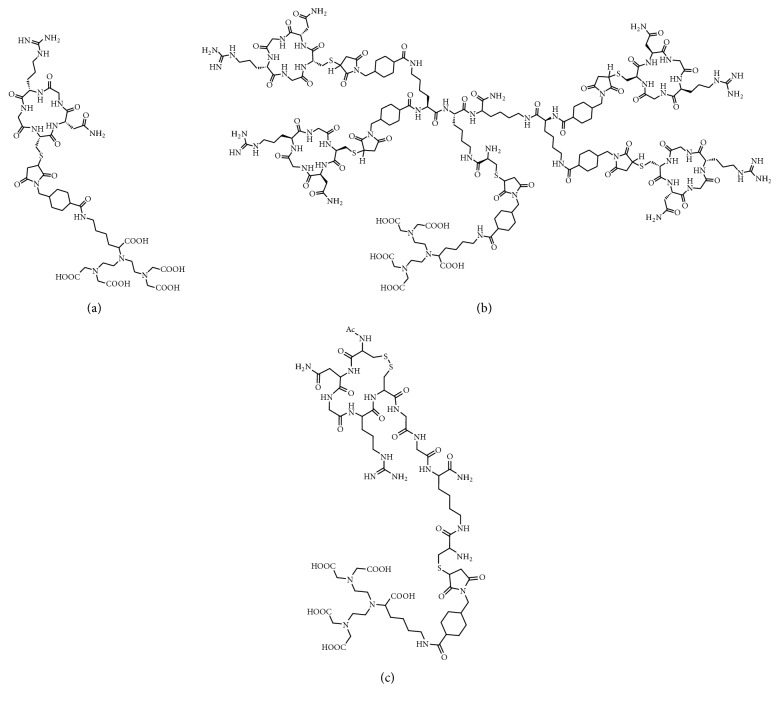
Structural formulas of DTPA-conjugated coNGR (a), co(NGR)_4_ (b), and cNGR (c).

Whereas the ring structure of our previously developed cyclic NGR peptide cyclic(NAc-Cys-Asn-Gly-Arg-Cys-Gly-Gly-Lys) was cyclized using a disulfide bond (henceforth called “cNGR”), we now cyclized the ring structure via a peptide bond by native chemical ligation (NCL, Figure 1) resulting in a new monomeric tracer: cyclo(CNGRG) (“coNGR”). Theoretically, this would lead to a more stable molecule and higher target uptake in vivo. Because a multimerization strategy has been shown to be beneficial for targeting and imaging with RGD peptides [16, 17], coNGR peptide moieties were coupled to a lysine wedge, resulting in a tetrameric coNGR imaging agent: cyclo(CNGRG)_4_ (“tetrameric coNGR” or “co(NGR)_4_”). Both monomeric coNGR and tetrameric coNGR were conjugated with diethylene triamine pentaacetic acid (DTPA, Figures 2(a) and 2(b)), radiolabeled with ^111^In, and in vitro and in vivo studies were performed. Using dual-isotope SPECT imaging, these new angiogenesis imaging agents were tested in a mouse model for MI in combination with the perfusion tracer ^99m^Tc-sestamibi.

## 2. Materials and Methods

### 2.1. Synthesis of coNGR and co(NGR)_4_

#### 2.1.1. Synthesis of Linear H-[Cys^1^-Asn^2^-Gly^3^-Arg^4^-Gly^5^]-MpaL-NH_2_

Boc-Cys(MeBzl)-OH, Boc-Asn(Xan)-OH, and Boc-Arg(Tos)-OH were purchased from Bachem (Bubendorf, Switzerland). Boc-Gly-OH was obtained from Peptide Institute, Inc. (Osaka, Japan).

Linear CNGRG-MpaL thioester peptide was synthesized on methylbenzhydrylamine- (MBHA-) polystyrene resin (ChemPep, Wellinton, FL, USA; 0.2–0.4 mmol scale) as described previously [18, 19]. Instead of 2-(1*H*-benzotriazole-1-yl)-1,1,3,3-tetramethyluronium hexafluorophosphate (HBTU), 2-(6-Chloro-1*H*-benzotriazol-1-yl)-1,1,3,3-tetramethylaminium hexafluorophosphate (HCTU, Peptides International, Louisville, KY, USA) was used as coupling reagent. After the peptide was cleaved from the resin by treatment with anhydrous HF (GHC Gerling, Holz & Co. Handels GmbH, Hamburg, Germany), the crude product was analyzed on a Waters™ (Milford, MA, USA, and Etten-Leur, The Netherlands) ultrahigh performance liquid chromatography mass spectrometry (UPLC-MS) XEVO-G2QToF system. The peptide was purified by semipreparative HPLC using Vydac C_18_ HPLC columns (10 mm × 250 mm, 12 mL/min flow rate or 22 mm × 250 mm, 20 mL/min flow rate; Grace Davison Discovery Sciences, Deerfield, IL, USA) connected to a Waters Deltaprep System consisting of a Waters Prep LC Controller and a Waters 2487 Dual wavelength Absorbance Detector (*λ* = 214 nm). To elute the peptide, an appropriate gradient of buffer B in buffer A, where buffer A is 0.1% trifluoroacetic acid (TFA, Biosolve BV, Valkenswaard, The Netherlands) in H_2_O/CH_3_CN (95/5, v/v, Biosolve) and buffer B is 0.1% TFA in CH_3_CN/H_2_O (90/10, v/v), was used.

#### 2.1.2. Cyclization of H-[C-N-G-R-G]-MpaL-NH_2_ by NCL

For cyclization, the peptide was dissolved in 50 mM (NH_4_)_2_CO_3_ pH 7.8 (Sigma Aldrich, Steinheim, Germany) at a maximum concentration of 1 mg/mL. The reaction was performed at 37°C and was followed over time by UPLC-MS analysis. Generally, cyclization was complete after 1 h. After cyclization, coNGR was purified as described above.

#### 2.1.3. co(NGR)_4_

To synthesize a tetrameric cyclic NGR peptide, a scaffold peptide with 4 NGR-coupling sites was necessary. Therefore, a lysine wedge was synthesized on MBHA resin (0.2 mmol scale). First, Boc-Lys(Fmoc)-OH (Bachem; Fmoc = 9-fluorenylmethoxycarbonyl) was coupled to the solid support, followed by two coupling cycles of Boc-Lys(Boc)-OH (Bachem). After chain assembly of the* N*^*α*^-Boc protected lysine wedge, 313 mg peptidyl-resin was treated with piperidine 20 v-% in DMF (4 × 3 min, both Biosolve) for* N*^*ε*^-Fmoc group removal. Then, Boc-thiazolidine-4-carboxylic acid (Thz; Bachem; 259 mg) was coupled using 2 mL 0.5 M HCTU and 400 *µ*l* N*,*N*-Diisopropylethylamine (DiPEA; Biosolve). After treatment of the peptidyl-resin with TFA (2 × 1 min) for* N*^*α*^-Boc-deprotection of the 4 lysine residues and coupling of succinimidyl 4-(*N*-maleimidomethyl)cyclohexane-1-carboxylate (SMCC; 1.47 g in 6 mL DMF), the peptidyl-resin was cleaved from the resin using HF. Subsequently, the crude Lys(Thz)-(Lys)_2_-(SMCC)_4_ product was purified on semipreparative RP-HPLC. For synthesis of the tetrameric coNGR peptide, 5.3 mg coNGR and 1.7 mg of functionalized lysine wedge were dissolved in 1 mL 0.1 M sodium phosphate buffer pH 6.5, containing 6 M GuHCl (Sigma Aldrich). After 3 h at 37°C, product formation was confirmed on UPLC-MS and the reaction mixture was purified on semipreparative HPLC as described above.

#### 2.1.4. Maleimide-DTPA Coupling

Maleimide-DTPA was prepared via the method described by Dirksen et al. [20, 21]. coNGR or co(NGR)_4_ and maleimide-DTPA (1.5 eq) were dissolved in 0.1 M acetate pH 4, MeONH_2_, EDTA, 6 M GuHCl. Both reactions were performed at 37°C and followed on UPLC-MS. After reaction completion, the reaction mixture was purified on semipreparative HPLC.

Analytical mass data of linear CNGRG-MpaL, coNGR, Lys(Thz)-(Lys)_2_-(SMCC)_4_, DTPA-coNGR, and DTPA-co(NGR)_4_ are given in Table 1.

### 2.2. Imaging Probes

#### 2.2.1. ^111^In-coNGR and ^111^In-co(NGR)_4_

coNGR and co(NGR)_4_ were radiolabeled with ^111^InCl_3_ (Mallinckrodt, Petten, The Netherlands) analogous to what has been described previously [15]. cNGR was used as reference compound and labeled with ^111^InCl_3_ as described previously [15].

#### 2.2.2. Stability Tests

^111^In-cNGR, ^111^In-coNGR, or ^111^In-co(NGR)_4_ was added to 1 × 10 mL human blood in heparin (BD Biosciences, Vianen, The Netherlands). Blood stability was tested at 0, 10, 30, and 50 min. For each time point imaging agents were separated from blood cells and proteins by adding 0.5 mL MeCN (VWR International BV, Breda, The Netherlands) to 0.5 mL blood sample followed by centrifugation (2,000 ×g, 3 min). Supernatant samples were analyzed by HPLC using the above-mentioned method.

#### 2.2.3. Octanol/Water Partition Coefficient

The log⁡*P* value of each compound was determined in three separate experiments as described previously [22].

#### 2.2.4. ^99m^Tc-Sestamibi

Freshly prepared ^99m^Tc-sestamibi (Technescan sestamibi) was ordered from GE Healthcare (Eindhoven, The Netherlands).

### 2.3. Animal Studies

In 10–12-week-old male Swiss mice, we induced MI by ligation of the left anterior descending coronary artery (LAD) as described before [23] to test coNGR (*n* = 5) or co(NGR)_4_ (*n* = 8). A group of sham-operated Swiss mice (*n* = 5) was used as control. SPECT imaging was performed 7 days after MI or sham surgery. An overview of the number of animals used per tracer is given in Table S1. All animals were held under the guidelines of the animal care facility (Maastricht University). All animal experiments were approved by the Committee for Animal Welfare of Maastricht University.

### 2.4. Micro-SPECT

Mice were anesthetized with isoflurane (induction 2.5%; maintenance 1.5%), a catheter was placed in a tail vein, and animals were positioned in the SPECT camera (MILabs, Utrecht, The Netherlands). Prior to image acquisition, a bolus injection of ^99m^Tc-sestamibi and ^111^In-coNGR or ^111^In-co(NGR)_4_ was given intravenously (i.v.) in a maximum volume of 200 *µ*L. Table S1 in Supplementary Material available online at https://doi.org/10.1155/2017/8638549 provides an overview of the average injected dose. Immediately after injection, 4 consecutive time frames of 15 min each were acquired. For image quantification we used the last of these frames.

### 2.5. SPECT Image Reconstruction

Acquired list mode data was reconstructed using MILabs reconstruction software (version 2.51) employing the POS-EM algorithm (6 iterations and 16 subsets, reconstructed at a voxel size of 0.4 mm). ^99m^Tc and ^111^In images were reconstructed by selecting photo peak and background energy levels as described before [15].

### 2.6. Image Quantification

To allow quantification of imaging agent uptake, in vivo isotope-specific conversion factors (CF) were determined for the 0.6 mm collimator in a representative phantom with a known activity. Using the previously described method [15] the following conversion factors (CF), CF_99mTc_ 612 MBq/mL and CF_111In_ 643 MBq/mL, were found.

PMOD 3.7 cardiac tool PCARD (PMOD technologies, Zürich, Switzerland) was used to segment the heart in the 17-segment model. Uptake per segment was subsequently expressed as a mean standardized uptake value (SUV_mean_), also using the previously described method [15]. For body weight, we assumed that 1 g equalled 1 mL.

### 2.7. Biodistribution

After imaging, the vital organs were harvested and kept for gamma-counting (Wallac Wizard, Turku, Finland). Acquired data were expressed as percentage injected dose per gram tissue (% ID/g).

### 2.8. CD13 Immunohistological Staining

Hearts were dissected and fixated in HEPES-buffered formaldehyde containing 150 mM saline for 24 h at 4°C. Hereafter, hearts were placed in 70% ethanol for maximal one month before embedding in paraffin. Paraffin-embedded hearts were cut at 4 *µ*m thickness. Sections were deparaffinized and rehydrated after which endogenous peroxidase was blocked by 0.3% H_2_O_2_ in methanol. After antigen retrieval (DAKO, target retrieval solution) sections were blocked in 5% goat serum for one hour. Next, sections were incubated overnight with primary monoclonal antibody against CD13 (1 : 500, Sigma). After washing, sections were incubated with BrightVision poly-horseradish peroxidase (HRP) Goat anti-Rabbit antibody (Immunologic, Duiven, The Netherlands). HRP was visualized by NOVAred substrate kit (VECTOR Laboratories Inc., Burlingame, CA, USA) and sections were counterstained with hematoxyline. Finally, sections were covered by cover glass with entellan and visualized using Leica Application Suite X (Leica Microsystems, Wetzlar, Germany).

### 2.9. Statistics

All data were expressed as mean ± SEM. To test for significant differences we performed an unpaired student's *t*-test with *P* < 0.05 being considered statistically significant. Data were analyzed using Microsoft Excel (version 2010).

## 3. Results and Discussion

In this study, an NGR peptide cyclized via native chemical ligation (NCL) and its tetravalent analog were designed and synthesized. The feasibility of these two new NGR peptide-based ligands for radionuclide imaging of CD13 expression in a mouse MI model with SPECT was explored. The peptides were radiolabeled with ^111^In and used for dual-isotope SPECT with ^99m^Tc-sestamibi as myocardial perfusion agent. Uptake of ^99m^Tc-sestamibi and ^111^In-coNGR and ^111^In-co(NGR)_4_ was quantified in the 17-segment model.

### 3.1. Chemistry

All observed masses of the constructs fell within the range of theoretical monoisotopic and average masses. Structural formulas of final products are given in Figures 2(a)–2(c) and HPLC chromatograms of ^111^In-labeled NGR peptides are displayed in Figure S1. Radiochemical purity generally exceeded 95% for coNGR, co(NGR)_4_, and cNGR and did not require further purification. From stability studies it appeared that disulfide bond-cyclized cNGR was less stable after 50 minutes of incubation in blood (7.5% intact product, Table 2) compared to both coNGR-based tracers. The percentage of intact tetrameric coNGR (25.3%) is slightly higher compared to coNGR (20.2%).

Octanol-water partition coefficients indicated that coNGR was more hydrophilic than co(NGR)_4_ (Table 3). Remarkably, the reference compound cNGR appeared initially to be the most lipophilic compound (−3.30 ± 0.14). However, over time its log⁡*P* value decreased soon to −4.56 ± 0.05 (<60 min), most likely explained by instability of this compound. Apparently, backbone cyclization via NCL resulted in a more stable ring structure than cyclization via disulfide bond formation.

### 3.2. SPECT and Analysis of coNGR and co(NGR)_4_ Uptake

**Figure 3:**
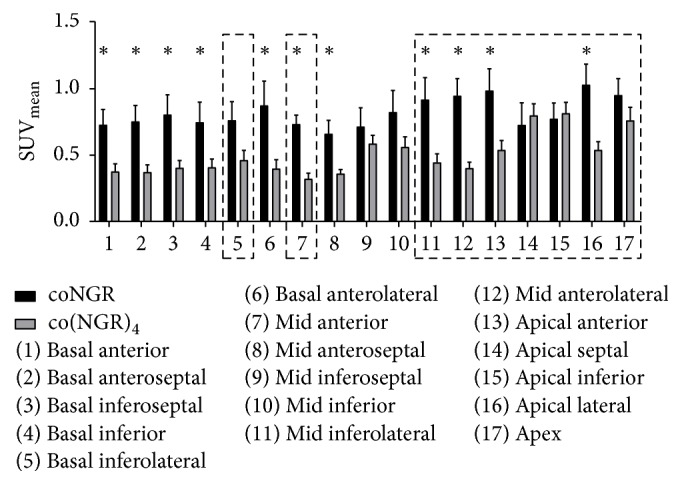
Overview of the SUVs for coNGR and co(NGR)_4_ in MI animals. Infarcted areas (significantly decreased ^99m^Tc-sestamibi uptake) in coNGR group or co(NGR)_4_ group are encirkeld by the dashed line. ^*∗*^*P* < 0.05 is considered statistically significant.

**Figure 4:**
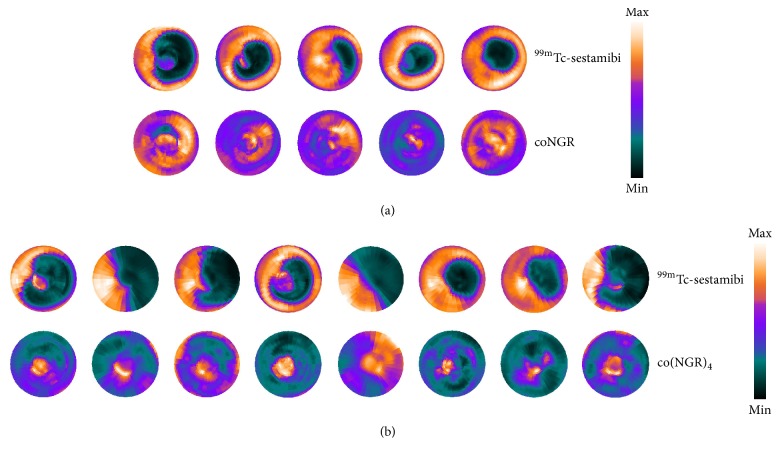
Polar perfusion maps from each mouse in the MI group displaying the uptake pattern of ^99m^Tc-sestamibi and coNGR (a) or ^99m^Tc-sestamibi and co(NGR)_4_ (b). The uptake in the polar perfusion maps is color coded and relative to the injected dose per animal.

**Figure 5:**
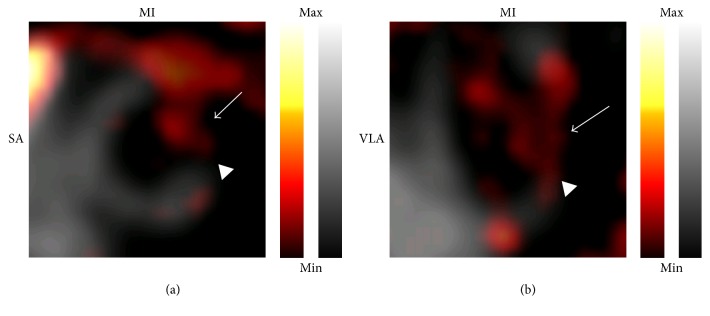
Representative in vivo fusion images of ^99m^Tc-sestamibi and coNGR uptake in MI mice. In “grey” is the uptake of ^99m^Tc-sestamibi in myocardium, while uptake of coNGR is in “hot metal” color. In (a) and (b) slices of an infarcted heart are shown. The infarct area is visualized by the decreased uptake of ^99m^Tc-sestamibi (in grey) which is clearly visible in the anterolateral region of the heart (arrowheads). Enhanced uptake of coNGR is clearly visible in the infarct area (arrows). Note: the uptake is color coded and relative to the injected dose per animal. SA: short axis; VLA: vertical long axis.

**Figure 6:**
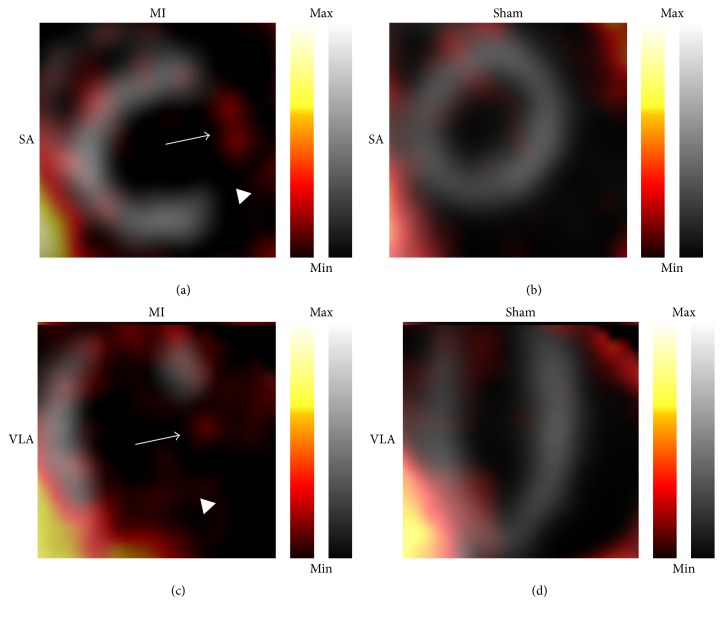
Representative in vivo fusion images of ^99m^Tc-sestamibi and co(NGR)_4_ uptake. In “grey” is the uptake of ^99m^Tc-sestamibi in myocardium, while uptake of co(NGR)_4_ imaging is in “hot metal” color. (a) and (c): infarcted heart. Infarct area is signified by decreased uptake of ^99m^Tc-sestamibi which is clearly visible in the anterolateral region of the heart (arrowheads). Enhanced uptake of co(NGR)_4_ is clearly visibly in the infarct area (arrows). (b) and (d): sham-operated heart. Uniform uptake of ^99m^Tc-sestamibi can be seen in combination with low overall uptake of co(NGR)_4_. Note: uptake is color coded and relative to injected dose per animal. SA: short axis; VLA: vertical long axis.

The 17-segment model was used to determine standardized uptake values (SUVs) of the tracers [24]. ^99m^Tc-sestamibi allowed visualization of infarcted myocardium 7 days after LAD ligation. Infarcted areas were defined as areas with significantly reduced uptake of ^99m^Tc-sestamibi in either coNGR or co(NGR)_4_ injected mice with LAD ligation compared to sham-operated mice (Table S2). Highest uptake of ^111^In-coNGR and ^111^In-co(NGR)_4_ was observed in the area in and around the apex, which corresponds to the infarct area (Figures 3 and 4 and Table S3). Uptake patterns of coNGR and co(NGR)_4_ can be seen in Figures 5 and 6, respectively.

To examine whether multimerization indeed resulted in higher target uptake, SUVs of co(NGR)_4_ and coNGR in infarcted and noninfarcted areas were compared for each tracer separately. Uptake of co(NGR)_4_ in segments that were affected by MI was significantly higher (0.57 ± 0.03) than in segments that were not affected by MI (0.43 ± 0.02), whereas uptake of coNGR in MI-affected segments (0.83 ± 0.05) was not significantly higher than in unaffected segments (0.76 ± 0.05). This suggests that co(NGR)_4_ has a more specific uptake in infarcted areas than coNGR.

Although coNGR and co(NGR)_4_ were not directly compared with cNGR in an in vivo study, both coNGR-based ligands showed a higher uptake than cNGR in the infarcted area. However, only co(NGR)_4_ indicated a more target-specific uptake which makes co(NGR)_4_ a more optimal agent than cNGR or coNGR for imaging of angiogenesis after MI.

### 3.3. Biodistribution of coNGR and co(NGR)_4_

**Figure 7:**
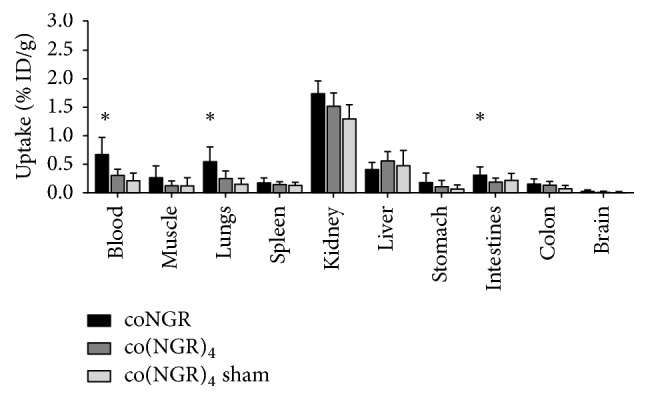
Biodistribution of coNGR and co(NGR)_4_ in MI mice and co(NGR)_4_ in sham-operated mice. Substantial kidney uptake of coNGR and co(NGR)_4_ was observed. coNGR had significantly higher uptake than co(NGR)_4_ in blood, lungs, and intestines whereas uptake in other organs was similar. ^*∗*^*P* < 0.05 is considered statistically significant.

One hour postinjection (p.i.), urinary excretion in MI mice was 72.5 ± 5.2 for coNGR and 54.9 ± 9.1 for co(NGR)_4_ (% ID/g ± SEM, *P* = 0.23). Retention of coNGR appeared to be significantly higher in blood, muscle, lungs, and intestines compared to retention of co(NGR)_4_. It could be that co(NGR)_4_ clears a bit more via the hepotobiliary route than coNGR, though liver uptake of co(NGR)_4_ and coNGR was not statiscally different. Other organs displayed a low uptake of coNGR that did not differ from the uptake of co(NGR)_4_ (Figure 7).

Unlike with cyclic RGD, cyclic NGR multimerization did not result in higher target uptake as coNGR exceeded co(NGR)_4_ uptake. Changing the currently used short SMCC spacer, which only allows statistical rebinding, for a longer and less rigid spacer that might enable binding to multiple CD13 receptors simultaneously, could improve the avidity and thereby the affinity of the tracer. For example, a flexible PEG spacer with a length that could bridge the width of the CD13 receptor of 131 Å [25] might be suitable.

### 3.4. CD13 Immunohistochemistry

**Figure 8:**
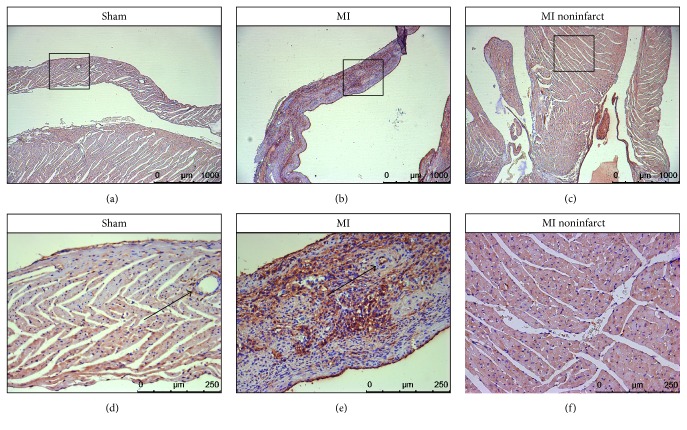
Representative pictures of CD13 expression in myocardium of sham and MI operated animals. Low baseline expression of CD13 was observed on photomicrographs (magnification 40x) of the myocardium of sham-operated animals (a). Dramatically increased CD13 expression was observed on photomicrographs (magnification 40x) of infarcted myocardium of MI operated animals (b). On photomicrographs (magnification 40x) of noninfarcted myocardium, a marginally higher expression of CD13 was noticed (c). Photomicrographs (magnification 200x) in (d), (e), and (f) represent a magnification of the region indicated in (a), (b), and (c), respectively. Note that expression of CD13 is not restricted to blood vessels but that endothelium of blood vessels is positive in the infarcted heart as opposed to the sham (arrows).

To validate uptake patterns of both coNGR-based tracers, cardiac CD13 expression was evaluated through immunohistological staining. A low level of CD13 expression was observed within the myocardium of sham-operated control animals (Figures 8(a) and 8(d)). While the level of CD13 expression in the noninfarcted myocardium of MI animals appeared marginally higher compared to the expression level in control animals (Figures 8(c) and 8(f)), the level of CD13 expression was markedly higher in the infarcted myocardium of MI animals (Figures 8(b) and 8(e)).

The enhanced uptake of coNGR and co(NGR)_4_ in the healthy myocardium of infarcted hearts points towards an overall angiogenic response of the heart in MI animals. Additionally, for co(NGR)_4_ the highest level of uptake was found in and around the infarcted areas of MI animals which also correlated to the histological findings as the highest level of CD13 expression was found in and around the infarcted areas. The same trend was observed for coNGR. It is highly likely that the uptake in the infarcted area as well as in the healthy myocardium is specific and related to increased CD13 expression.

## 4. Conclusions

Two CD13-targeting SPECT tracers for angiogenesis, coNGR and co(NGR)_4_, were designed and synthesized. However, target uptake of cyclic NGR-based imaging agents does not seem to increase with increasing valency. Instead, the key to enhance cyclic NGR-based imaging agent uptake was to stabilize the ring structure through NCL. Additional studies with different linkers conjugated to the lysine scaffold are warranted to investigate a possible increased avidity effect with coNGR-based imaging agents.

## Supplementary Material

Figure S1. Radio-HPLC chromatograms for ^111^In-labeled coNGR (a), co(NGR)_4_ (b), and the previously examined cNGR imaging agent (c). Radiochemical purity of all three tracers generally exceeded 95% and did not require further purification.
Table S1. Overview of the number of animals used per imaging agent. Injected activity is displayed as mean ± SEM. ∗A variable number of animals was available for biodistribution experiments.
Table S2. Overview of SUVs of ^99m^Tc-sestamibi in the coNGR and co(NGR)_4_ group. Data are displayed as mean ± SEM. ∗*p* < 0.05 is considered statistically significant from SUVs in sham operated animals.
Table S3. Overview of SUVs of coNGR and co(NGR)_4_ in MI mice. Data are displayed as mean ± SEM.

## Figures and Tables

**Table 1:** Analytical mass data of coNGR-based tracer constructs. Masses are given in Da. From top to bottom, mass of linear peptide (thioester), cyclic peptide after NCL, and the lysine wedge with four SMCC linkers are represented. Last two rows represent mass of DTPA-coNGR and DTPA-co(NGR)_4_, respectively.

Compound	Monoisotopic mass	Molecular weight	Measured
CNGRG-MpaL	705.31	705.81	705.32
coNGR	487.20	487.54	487.22
Lys(Thz)-(Lys)_2_-(SMCC)_4_	1520.77	1521.84	1520.83
DTPA-coNGR	1170.50	1171.25	1170.49
DTPA-co(NGR)_4_	4140.86	4143.69	4140.76

**Table 2:** Blood stability of ^111^In-labeled cyclic NGR-based SPECT tracers coNGR, co(NGR)_4_, and cNGR [15] after 50 min of blood incubation.

Tracer	Intact product (%)
coNGR	20.22
co(NGR)_4_	25.31
cNGR	7.55

**Table 3:** log⁡*P* values of ^111^In-labeled coNGR, co(NGR)_4_, and cNGR [15]. log⁡*P* values were determined in three separate experiments, hence indicated as log⁡*P*1, log⁡*P*2, and *P*3. *N* = 3 for each measurement.

Tracer	log⁡*P*_1_	log⁡*P*_2_	log⁡*P*_3_
coNGR	−4.57 ± 0.06	−4.60 ± 0.08	−4.54 ± 0.09
co(NGR)_4_	−3.65 ± 0.02	−3.64 ± 0.03	−3.84 ± 0.04
cNGR	−3.30 ± 0.14	−4.17 ± 0.01	−4.56 ± 0.05
