# Supplementary material for: Use of Cyclic Backbone NGR-Based SPECT to Increase Efficacy of Postmyocardial Infarction Angiogenesis Imaging
Source: Contrast Media Mol Imaging. 2017 Oct 24;2017:8638549. doi: 10.1155/2017/8638549 (PMC5674494; doi:10.1155/2017/8638549)
Supplement: Supplementary file 1 — Figure S1. Radio-HPLC chromatograms for 111In-labeled coNGR (a), co(NGR)4 (b), and the previously examined cNGR imaging agent (c). Radiochemical purity of all three tracers generally exceeded 95% and did not require further purification. Table S1. Overview of the number of animals used per imaging agent. Injected activity is displayed as mean ± SEM. ∗A variable number of animals was available for biodistribution experiments. Table S2. Overview of SUVs of 99mTc-sestamibi in the coNGR and co(NGR)4 group. Data are displayed as mean ± SEM. ∗p < 0.05 is considered statistically significant from SUVs in sham operated animals. Table S3. Overview of SUVs of coNGR and co(NGR)4 in MI mice. Data are displayed as mean ± SEM. [file 8638549.f1.docx]

**Supplemental Data**

**Figure S1**


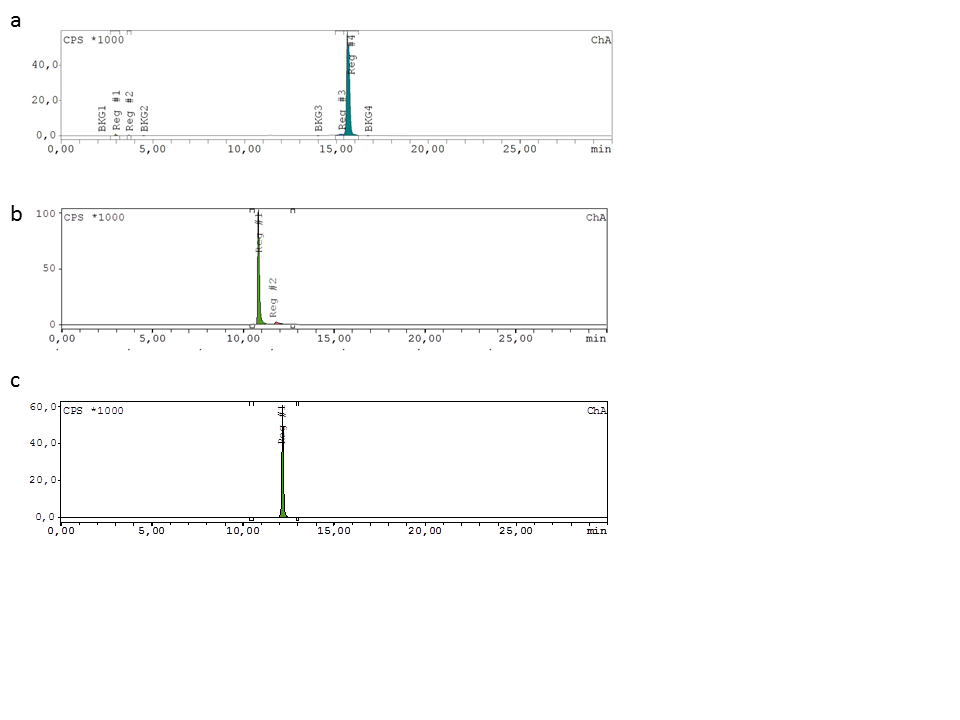


(c)

(b)

(a)

**Figure S1.** Radio-HPLC chromatograms for ^111^In-labeled coNGR (a), co(NGR)_4_ (b), and the previously examined cNGR imaging agent (c). Radiochemical purity of all three tracers generally exceeded 95% and did not require further purification.

**Table S1**

| **Procedure** | **Angiogenesis tracer** | **Injected dose**  **(MBq)** | **^99m^Tc-sestamibi (MBq)** | **SPECT** | **Bio-distribution** |
| --- | --- | --- | --- | --- | --- |
| MI | co(NGR)_4_ | 38,5 ± 1,3 | 28,6 ± 5,6 | N = 8 | N = 8 |
| Sham | co(NGR)_4_ | 47,3 ± 2,2 | 56,8 ± 8,2 | N = 6 | N ≥ 5* |
| MI | coNGR | 57,4 ± 0,9 | 48,0 ± 2,0 | N = 5 | N ≥ 3* |

**Table S1.** Overview of the number of animals used per imaging agent. Injected activity is displayed as mean ± SEM. *A variable number of animals was available for biodistribution experiments.

**Table S2**

|  | **Standardized uptake values (SUV) of ^99m^Tc-sestamibi** | | |
| --- | --- | --- | --- |
|  | **co(NGR)_4_ group** | | **coNGR group** |
| **Segments** | **Sham (n = 6)** | **MI (n = 8)** | **MI (n = 5)** |
| 1. Basal anterior | 2.91 ± .026 | 2.60 ± 0.70 | 2.46 ± 0.45 |
| 2. Basal anteroseptal | 2.72 ± 0.32 | 3.68 ± 0.42 | 2.28 ± 0.39 |
| 3. Basal inferoseptal | 2.89 ± 0.33 | 3.97 ± 0.49 | 2.33 ± 0.38 |
| 4. Basal inferior | 3.07 ± 0.33 | 3.06 ± 0.54 | 2.60 ± 0.56 |
| 5. Basal inferolateral | 3.03 ± 0.30 | 1.66 ± 0.47* | 2.23 ± 0.63 |
| 6. Basal anterolateral | 2.89 ± 0.20 | 1.60 ± 0.55 | 2.14 ± 0.52 |
| 7. Mid anterior | 3.00 ± 0.22 | 1.35 ± 0.31* | 1.41 ± 0.28* |
| 8. Mid anteroseptal | 3.10 ± 0.28 | 3.56 ± 0.47 | 2.46 ± 0.39 |
| 9. Mid inferoseptal | 3.16 ± 0.26 | 3.57 ± 0.68 | 2.27 ± 0.53 |
| 10. Mid inferior | 3.03 ± 0.29 | 2.21 ± 0.52 | 1.64 ± 0.67 |
| 11. Mid inferolateral | 3.06 ± 0.34 | 0.68 ± 0.07* | 0.75 ± 0.24* |
| 12. Mid anterolateral | 2.77 ± 0.22 | 0.62 ± 0.07* | 0.42 ± 0.09* |
| 13. Apical anterior | 2.93 ± 0.22 | 1.04 ± 0.25* | 0.71 ± 0.30* |
| 14. Apical septal | 3.23 ± 0.24 | 2.68 ± 0.30 | 1.67 ± 0.39* |
| 15. Apical inferior | 2.88 ± 0.32 | 2.06 ± 0.24 | 1.30 ± 0.31* |
| 16. Apical lateral | 2.44 ± 0.20 | 0.67 ± 0.11* | 0.59 ± 0.26* |
| 17. Apex | 2.71 ± 0.25 | 1.25 ± 0.32* | 0.98 ± 0.35* |

**Table S2.** Overview of SUVs of ^99m^Tc-sestamibi in the coNGR and co(NGR)_4_ group. Data are displayed as mean ± SEM. *p<0.05 is considered statistically significant from SUVs in sham operated animals.

**Table S3**

| **Standardized uptake values (SUV) of cNGR imaging agents** | | |
| --- | --- | --- |
|  | **coNGR** | **co(NGR)_4_** |
| **Segments** | **MI (n = 5)** | **MI (n = 8)** |
| 1. Basal anterior | 0.73 ± 0.12 | 0.37 ± 0.06 |
| 2. Basal anteroseptal | 0.75 ± 0.12 | 0.37 ± 0.05 |
| 3. Basal inferoseptal | 0.81 ± 0.15 | 0.40 ± 0.05 |
| 4. Basal inferior | 0.75 ± 0.15 | 0.41 ± 0.06 |
| 5. Basal inferolateral | 0.76 ± 0.14 | 0.46 ± 0.07 |
| 6. Basal anterolateral | 0.88 ± 0.18 | 0.40 ± 0.07 |
| 7. Mid anterior | 0.73 ± 0.07 | 0.32 ± 0.04 |
| 8. Mid anteroseptal | 0.66 ± 0.10 | 0.36 ± 0.03 |
| 9. Mid inferoseptal | 0.72 ± 0.14 | 0.59 ± 0.06 |
| 10. Mid inferior | 0.83 ± 0.16 | 0.56 ± 0.08 |
| 11. Mid inferolateral | 0.92 ± 0.17 | 0.44 ± 0.07 |
| 12. Mid anterolateral | 0.95 ± 0.13 | 0.40 ± 0.05 |
| 13. Apical anterior | 0.99 ± 0.17 | 0.54 ± 0.07 |
| 14. Apical septal | 0.73 ± 0.17 | 0.80 ± 0.09 |
| 15. Apical inferior | 0.77 ± 0.12 | 0.82 ± 0.08 |
| 16. Apical lateral | 0.96 ± 0.10 | 0.54 ± 0.06 |
| 17. Apex | 0.95 ± 0.13 | 0.76 ± 0.10 |

**Table S3.** Overview of SUVs of coNGR and co(NGR)_4_ in MI mice. Data are displayed as mean ± SEM.
